# Electrochemical Reduction of CO_2_ to C1 and C2 Liquid Products on Copper-Decorated Nitrogen-Doped Carbon Nanosheets

**DOI:** 10.3390/nano13010047

**Published:** 2022-12-22

**Authors:** Munzir H. Suliman, Zain H. Yamani, Muhammad Usman

**Affiliations:** Interdisciplinary Research Center for Hydrogen and Energy Storage (IRC-HES), King Fahd University of Petroleum & Minerals (KFUPM), Dhahran 31261, Saudi Arabia

**Keywords:** CO_2_ conversion, electrocatalysts, copper catalysts, carbon materials, ECO_2_RR

## Abstract

Due to the significant rise in atmospheric carbon dioxide (CO_2_) concentration and its detrimental environmental effects, the electrochemical CO_2_ conversion to valuable liquid products has received great interest. In this work, the copper-melamine complex was used to synthesize copper-based electrocatalysts comprising copper nanoparticles decorating thin layers of nitrogen-doped carbon nanosheets (Cu/NC). The as-prepared electrocatalysts were characterized by XRD, SEM, EDX, and TEM and investigated in the electrochemical CO_2_ reduction reaction (ECO_2_RR) to useful liquid products. The electrochemical CO_2_ reduction reaction was carried out in two compartments of an electrochemical H-Cell, using 0.5 M potassium bicarbonate (KHCO_3_) as an electrolyte; nuclear magnetic resonance (^1^H NMR) was used to analyze and quantify the liquid products. The electrode prepared at 700 °C (Cu/NC-700) exhibited the best dispersion for the copper nanoparticles on the carbon nanosheets (compared to Cu/NC-600 & Cu/NC-800), highest current density, highest electrochemical surface area, highest electrical conductivity, and excellent stability and faradic efficiency (FE) towards overall liquid products of 56.9% for formate and acetate at the potential of −0.8V vs. Reversible Hydrogen Electrode (*RHE)*.

## 1. Introduction

Recently, intensive fossil usage (such as coal, petroleum, and natural gas) is considered globally as the major energy source and has led to a dramatic increase in CO_2_ emission [1,2]. The present CO_2_ level is greater than 414 ppm [3]. Therefore, great efforts for the capture, sequestration, and utilization of CO_2_ should be devoted. Several techniques including biochemical, thermal, and electrochemical methods, have been extensively studied for their potential to convert CO_2_ into valuable chemicals [4,5]. The electrochemical CO_2_ reduction (ECO_2_RR) draws substantial attention due to its several advantages. In ECO_2_RR, the conversion process is controlled by the applied potential in the process [6]. The process also operates with electricity at ambient conditions, resulting in zero carbon emission. However, the ECO_2_RR required relatively high energy due to the stability of the CO_2_ molecule in an aqueous electrolyte. In order to lower the energy barrier and improve the performance and selectivity, an effective and long-lasting electrocatalyst is needed [7,8].

In the previous few years, several transition metals catalysts have been investigated, such as (Cu, Co, Zn, Sn, Ni, Bi, etc.) [9,10,11,12,13,14], bi-metallic (Cu-Zn, Cu-Ag, Cu-Sn, etc.) [15,16,17,18], oxides (CuOx, CuO-ZnO, etc.) [19,20], metal-organic frameworks [21,22,23,24], and zeolites [25,26]. Carbon-based electrocatalysts showed additional benefits beyond those already described, including low cost and availability, high electrical conductivity, and a large surface area that allows for the even distribution of active sites and the efficient adsorption of reactants [27,28,29]. Additionally, when nitrogen is doped in the carbon, the electrical conductivity is improved, and CO_2_ molecules are drawn to the catalyst’s surface more readily [30,31,32,33,34,35]. Han and co-authors claimed in their reports the synergy between the active sites of the Cu NPs and the N terminals in the supports facilitate the coupling of the CO (produced in the N-sites) and secondary (C), which lead to the formation of higher carbon alcohols products [36]. Recently, Bhunia et al. [31] reported the production of several liquid products with FE of 54 % at a potential of 1.0 V_RHE_ using Cu NPs supported on N-doped graphene. The selective ethanol production was also reported by Wang et al. [30], using N-doped carbon nanospikes decorated by the Cu NPs; this catalyst exhibited FE of 63% at a potential of 1.2 V_RHE_. Zhou et al. synthesized Cu@Cu_2_O coated with N-doped carbon derived from Cu-BTC MOF. The reported electrocatalysts showed 45% FE toward methanol production at −0.7 V potential [37].

**Figure 1:**
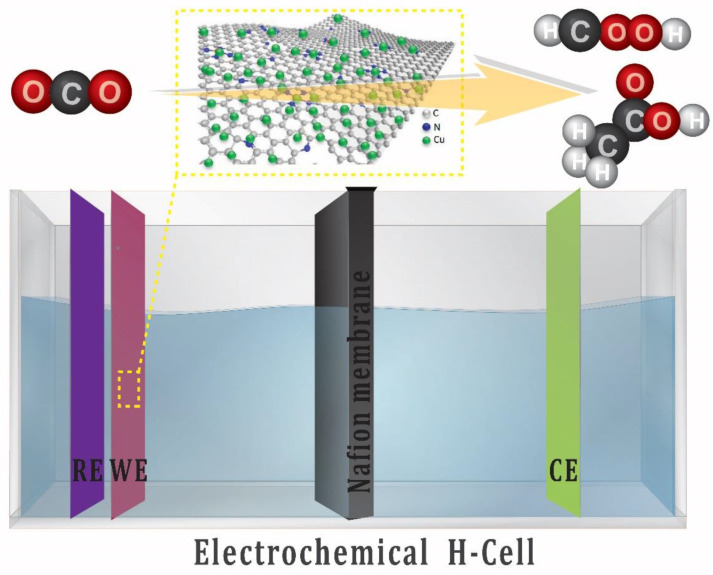
Schematic presentation of the three-electrode setup (H-Cell) for electrochemical CO_2_ reduction.

This work involved the fabrication of copper nanoparticle-decorated nitrogen-doped carbon nanosheets. The copper precursor for this electrocatalyst was complexed with a cheap organic linker (melamine), and then the resulting complex was pyrolyzed at various temperatures to produce the final electrocatalyst. After pyrolysis, small and evenly scattered Cu-NPs are formed due to the complexation of copper with melamine, which aids in the homogenous dispersion of copper atoms. Yuan and co-workers [38] reported the use of melamine crosslinked with 1-hydroxyethylidene-1,1-diphosphonic acid and some transition metal to form core-shell transition metal phosphides in N-doped carbon for water electrolysis and zinc air battery applications. However, in this study, the condition is optimized and melamine is directly crosslinked with the metal (Cu). The as-prepared Cu NPs/NC is used for the ECO_2_RR in H-Cell for the production of liquid products, as shown in Figure 1.

## 2. Experimental

### 2.1. Materials

Copper chloride dihydrate (CuCl_2_.2H_2_O) (99.95%) and melamine (99.0%) were purchased from Sigma Aldrich, US. Methanol (CH_3_OH) (99.8%) and diethyl ether ((CH_3_CH_2_)_2_O) (99.9%) were procured from Sharlu (Sharjah, United Arab Emirates). Nitrogen gas (N_2_) was supplied by Abdullah Hashem Industrial Gas Co., Ltd., Dammam, Saudi Arabia.

### 2.2. Preparation of Copper Melamine Complex

A total of 170 mg copper chloride dihydrate was dissolved in 20 mL of N_2_-purged methanol, then melamine (250 mg) was added to the solution. The mixture was heated to 100 °C for 14 h. After that, the solution was kept to cool at room temperature. The green powder was collected, washed three times with diethyl ether, and dried under vacuum at 50 °C.

### 2.3. Preparation of Copper Nanoparticles Decorated on Thin Carbon Nanosheets

The as-prepared copper melamine complex was placed in crucible and heated under N_2_ atmosphere at different temperatures (600, 700 and 800 °C) with 5 °C/min heating rate for 2 h to obtain Cu-NP/NC.

### 2.4. Preparation of Electrocatalyst

**Figure 2:**
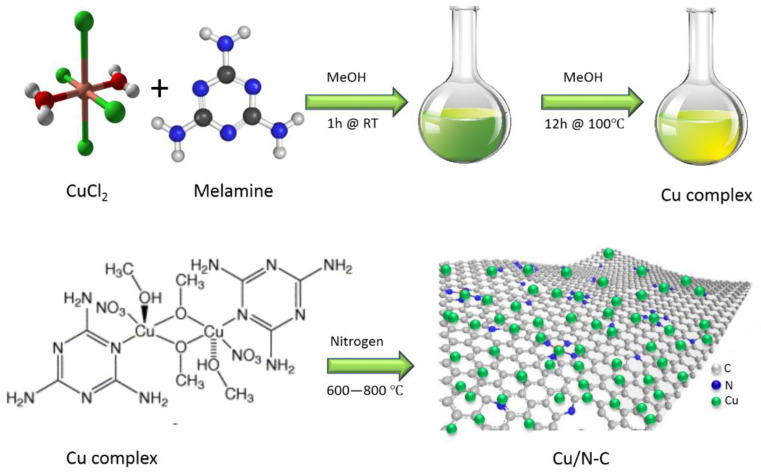
The synthesis schematic of copper-decorated nitrogen-doped carbon nanosheets.

10 mg of the Cu-NP/NC catalyst was dispersed in 1 mL mixture of 750 µL isopropanol, 200 µL DI water and 50 µL Nafion (5%). The mixture was sonicated for 20 min. Then 100 µL of the suspension was drop casted onto 1 cm^2^ conductive carbon paper and dried at room temperature. This preparation method is schematically presented in Figure 2.

### 2.5. Characterization

Morphological and detailed microstructural attributes of the materials were discerned by transmission and high-resolution transmission electron microscopy techniques (TEM/HR-TEM, Tecnai TF20) and field emission scanning electron microscopy (FESEM, Tescan Lyra-3). Other techniques employed for the characterization of the samples were X-ray diffraction (XRD, Rigaku MiniFlex) and ^1^H NMR spectroscopy (LAMBDA 500 spectrophotometer). Potentiostat (Gammray 620) was used for electrochemical analysis.

### 2.6. The Electrochemical Studies

The ECO_2_RR performance is investigated with an H-cell system consisting of a sliver silver chloride electrode (*Ag/AgCl*) as a reference electrode. A platinum mesh was used as a counter electrode. The as-prepared Cu-NP/NC film on conductive carbon paper was used as working electrode. A potentiostat (Gammray 620) is connected to the electrodes in the H-Cell. The ECO_2_RR performance was evaluated by carrying out linear sweep voltammetry (LSV) techniques and calculated the overpotential at different current densities (current normalized to the geometric surface area of the electrode). The cyclic voltammetry (CV) and LSV experiments were performed in 0.5 M potassium bicarbonate (KHCO_3_). All the electrochemical measurements were normalized to the *RHE* by using the following formula:

$$E_{RHE}=E_{Ag/AgCl}+0.059\times pH+E_{Ag/AgCl}^{\;}$$

where $E_{Ag/AgCl}^{\;}=0.199\;V$ [17].

The potential was swept from 0.0 to −1.4 V vs. *RHE*. The electrochemical impedance spectroscopy (EIS) was performed by varying the frequency from 10^5^ to 0.1 Hz under identical electrolyte and electrodes to the LSV.

The reduction products were evaluated by running the potentiostatic measurements at different potentials (−0.5 to −1.2) for 2 h, the liquid products were collected from the cell and quantified with ^1^H NMR.

## 3. Results and Discussion

**Figure 3:**
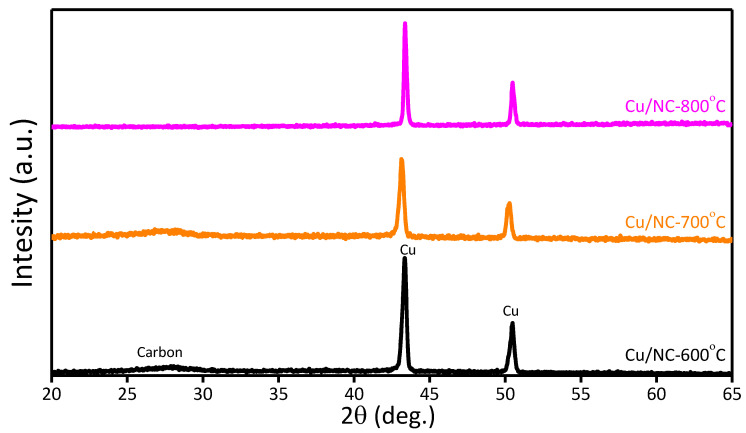
XRD of Cu-NP/NC.

The phase structure of Cu-NP/NC was investigated with powder XRD as shown in Figure 3. For the three catalysts Cu-NP/NC-600, Cu-NP/NC-700, and Cu-NP/NC-800, reflections at 43.4° and 50.3° were recorded ascribed for the planes (111) and (200), respectively (JCPDS number 01-085-1326) [39]. Additionally, the reflection peak at 26.2° (002) corresponded to NC (JCPDS# 03-065-6212) [40], which indicates the successful formation of metallic copper of nitrogen-doped carbon.

Further information about the composites’ chemical composition was explored using the EDS (Figure S1), which confirms the existence of the elements (Cu, C and N). Therefore, from the XRD and the EDS, the formation of metallic copper on nitrogen-doped carbon was confirmed.

**Figure 4:**
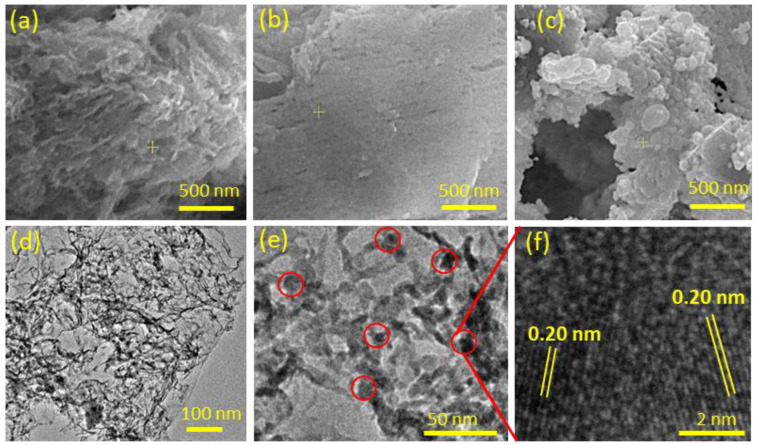
SEM of (**a**) Cu-NP/NC-600, (**b**) Cu-NP/NC-700, (**c**) Cu-NP/NC-700, (**d**,**e**) TEM of Cu-NP/NC-700 and (**f**) HRTEM of Cu-NP/NC-700.

The microstructure and morphology of the Cu-NP/NC were inspected with the SEM and the TEM. Figure 4a shows the SEM image of Cu-NP/NC-600, which reveals sheet-like morphology and the showed a thin sheet in the case of Cu-NP/NC-700 (Figure 4b). However, upon increasing the temperature to 800 °C the copper particles start to grow and agglomerate, as it is observed in Figure 4c for the catalyst Cu-NP/NC-800. The TEM (Figure 4d) confirms the formation of small and uniform dispersed copper nanoparticles (<20 nm) onto the thin sheet of carbon. The copper nanoparticles were smaller than 10 nm in size (Figure 4e), and in Figure 4f the high-resolution TEM (HRTEM) was carried out for the highlighted particles and the interplanar distance was estimated to be 0.2 nm, corresponding to the phase (111) for the metallic copper.

**Figure 5:**
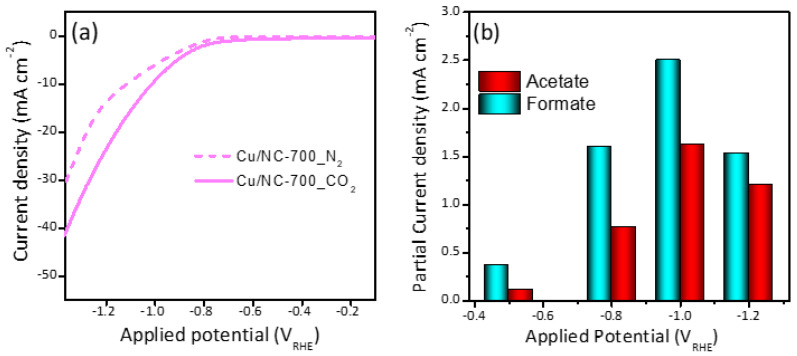
(**a**) LSV curves of Cu-NP/NC-700 electrocatalysts in N_2_ and CO_2_ saturated 0.5 M KHCO_3_ electrolyte. (**b**) Partial current densities of Cu-NP/NC-700.

The LSV was recorded for the three electrocatalysts in CO_2_ saturated 0.5 M KHCO_3_ and compared with N_2_ saturated in the same electrolyte as shown in Figure 5a. The polarization curves were demonstrated in Figure S2, which shows that the current density (CD) was increasing with increasing potential. It can be noted that there is significant enhancement upon the saturation of the electrolyte with CO_2_ (solid lines) compared to N_2_ (dashed lines). The observed CD at a potential of 1.0 V_RHE_ was −10, −6.2, and −3.7 mA cm^−2^ for the electrocatalysts Cu-NP/NC-700, Cu-NP/NC-600 and Cu-NP/NC-800, respectively. This activity order could be explained as follows: for the sample prepared at 600 °C less graphitic carbon and nitrogen were formed compared to the catalyst Cu-NP/NC-700, which significantly influences the electrode’s conductivity. However, the electrode Cu-NP/NC-800 preparation of the sample at a higher temperature led to higher degree of agglomeration as observed in the SEM (Figure 2c), which led to a drop in the surface area and accordingly decrease in the electrochemical performance. The partial current densities were shown in Figure 5b, which is the current required to generate formate and acetate. It can be observed that the partial current density is increasing with an increase the potential until −1.0 V. Moving to more cathodic potential (−1.2 V), the partial current densities of acetate and formate decreased. 

**Figure 6:**
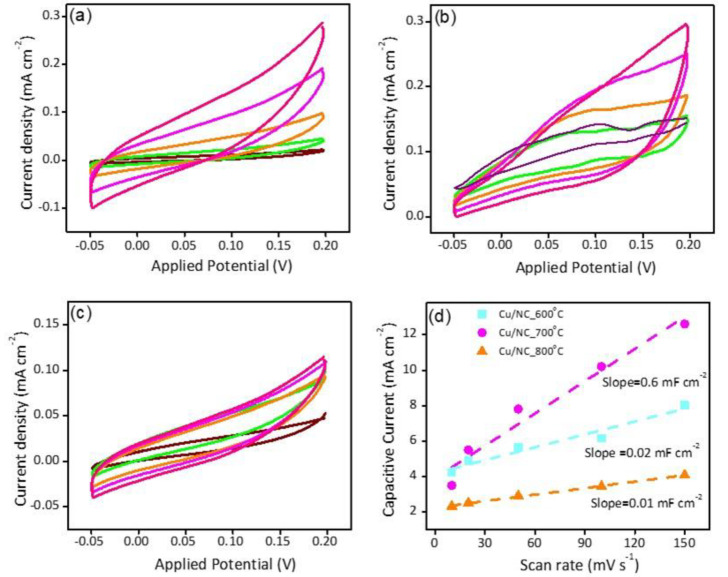
CVs of (**a**) Cu-NP/NC-600, (**b**) Cu-NP/NC-700, (**c**) Cu-NP/NC-800 electrocatalysts in CO_2_ saturated 0.5 M KHCO_3_ electrolyte, and (**d**) is the respected C_dl_ slopes of the electrodes. The scan rate (a to c) purple: 50 mVs^−1^, Green: 100 mVs^−1^, Orange: 150 mVs^−1^, Magenta: 200 mVs^−1^, Pink: 250 mVs^−1^.

Electrochemical surface area (ECSA) was estimated by calculating the double layer capacitance (C_dl_) [28,41]. Figure 6a–c shows the recorded CVs in the capacitive region for the electrodes Cu-NP/NC-600, Cu-NP/NC-700, and Cu-NP/NC-800, respectively. Figure 6d shows the respective slopes calculated from the previous figures which represent the Cdl. The electrode Cu-NP/NC-800 exhibited the lower C_dl_ (0.1 mF cm^−2^) due to the agglomeration of the Cu particles, followed by Cu-NP/NC-600 (0.2 mF cm^−2^) and finally, the electrocatalyst Cu-NP/NC-700, which possessed the highest ECSA

**Figure 7:**
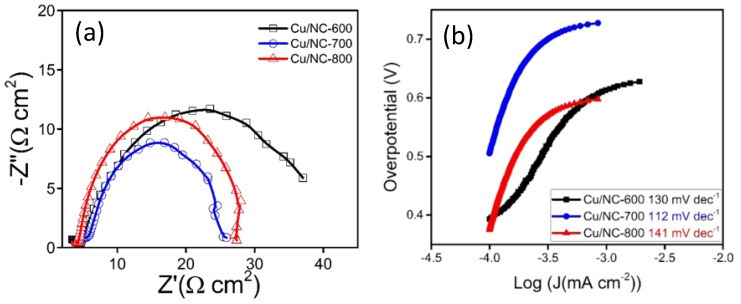
(**a**) Nyquist plots of Cu-NP/NC electrodes in CO_2_ saturated 0.5 M KHCO_3_ electrolyte. (**b**) Tafel slopes for the Cu/NC electrodes.

With the highest Cdl (0.6 mF cm^−2^). Moreover, the electrode conductivity is considered as a critical factor in the electrochemical performance; hence, the conductivity of the catalysts was investigated with the electrochemical impedance spectroscopy (EIS). EIS is a very important tool used to understand the electrode conductivity and the charge transfer resistance (Rct). The Nyquist plot is obtained from the EIS experiment; the smaller semicircle represents the higher conductivity. Figure 7a reveals the Nyquist plot for the three electrodes at applied potential of −1.0 vs. *RHE*. The Rct values were 31.5, 26.0, and 27.5 Ω cm^2^ for the electrodes Cu-NP/NC-600, Cu-NP/NC-700, and Cu-NP/NC-800, respectively. As expected, the sample prepared at 700 °C with less degree of agglomeration with graphitic carbon and nitrogen exhibited the highest conductivity (lower Rct).

**Figure 8:**
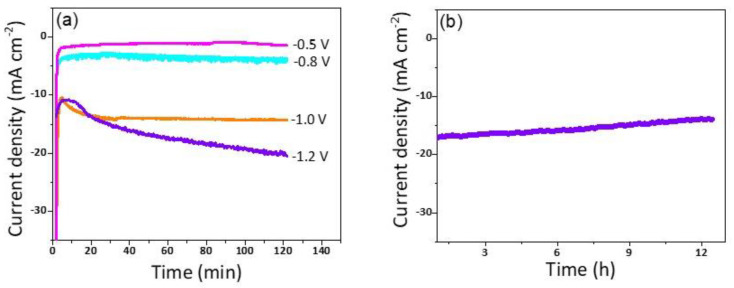
(**a**) The chronoamperometry of Cu/NC-700 at potentials of −0.5, −0.8, −1.0, and −1.2 V vs *RHE* for 2 h. (**b**) The long-term stability of Cu/NC-700 at potentials of −1.0 V vs *RHE* for 12 h.

**Figure 9:**
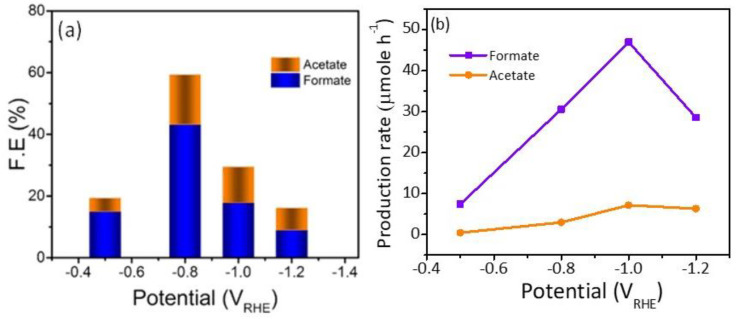
(**a**) FEs. (**b**) Production rate of the liquid products by Cu/NC-700 at the function of potential.

To obtain an idea about the kinetics and the mechanism of the reduction reaction Tafel slopes were investigated and compared for the three Cu/NC electrodes. Tafel slopes were estimated for the three electrodes using Tafel plots (Figure 7b). From the figure, the estimated values were 130, 112, and 141 mV dec^−1^ for the electrodes Cu/NC-600, Cu/NC-700, and Cu/NC-800, respectively. The Tafel equation suggests that the smaller slope value is translated into faster reaction kinetics [42]. The Cu/NC-700 exhibited the lowest Tafel value with an excellent agreement with values reported in the literature for Cu-based electrocatalysts. This small value suggests facilitated activation of the adsorbed CO_2_ on the surface of the catalyst (by the stabilization of the CO_2_^•**—**^). Additionally, it has been reported that the N atom doped in the carbon is considered as an excellent active site for CO production due to its weak adsorption energy, which led to the desorption of CO [36]. Moreover, the stability of the Cu/NC-700 was investigated using chronoamperometry by applying constant potential for a period of time and recording the produced current density. As it is observed in Figure 8b, the electrode Cu/NC-700 exhibited excellent stability at −15 mA cm^−2^ for 12 h in CO_2_ saturated in 0.5 M KHCO_3_ with no significant drop in the current. Chronoamperometry was carried out at different potentials (−0.5, −0.8, −1.0 and −1.2 V) for 2 h (Figure 8a), then after the chrono, the solution was evaluated using ^1^H NMR (Figure S3). As in Figure 9, two conversion products were observed: formic and acetic acid. The highest FE of 59.1% was a conversion rate of 31.0 and 3.2 µmol h^−1^ for the formic acid and acetic acid, respectively. The higher cathodic current observed at higher potential is predominant by the hydrogen evolution reaction (HER), obtained from the water reduction [4]. 

The electrochemical performance and conversion efficiency were compared with recent similar reports in Table 1, which compares other Cu-based, carbon based, and Cu-carbon composites for the electroreduction of CO_2_ into useful liquid products. The Cu/NC-700 can produce formic acid with a FE of 40.9% at low potential of −0.8 V vs. *RHE*. Acetic acid can also be significantly detected with a FE of 16% is in the range of the reported literature.

The proposed mechanism for CO_2_ reduction using this catalyst is as follows: firstly adsorption and reduction of CO_2_ (to the catalyst surface) to form CO_2_ radical (CO_2_^•**—**^). The formed radical is got protonated by the electrolyte to form (HCOO**^—^**)_ads_, which desorb to generate formate. In the case of acetate, prior to the protonation step of the first (CO_2_^•**—**^) radical, a second (CO_2_^•**—**^) radical is combined with first one to form (**^—^**OOC—COO**^—^**)**;** similarly, this intermediate is protonated and forms acetate. Since the yield of formate is higher than acetate, this means the rate of (CO_2_^•**—**^) protonation is faster than the rate of (**^—^**OOC—COO**^—^**) formation [43,44].

## 4. Conclusions

In this work, N-doped carbon nanosheets supported copper nanoparticles (Cu/NC) were prepared via pyrolysis of copper melamine complex at different temperatures and were investigated for the electrochemical CO_2_ reduction reaction in 0.5 M KHCO_3_ solution. The Cu/NC-700 exhibited the highest current density and selectivity for the conversion of CO_2_ with faradic efficiencies of 43.2% for formic acid and 16.1% for acetic acid, with a conversion rate of 34.0 and 3.2 µmol h^−1^, respectively at a reduction potential of −0.8 V vs. *RHE* and a current density of −4.9 mA cm^−2^. Moreover, the optimized electrocatalyst shows long term stability without significant loss in current density for 12 h. The Cu/NC-700 electrode exhibited a higher ECSA than Cu/NC-600 and Cu/NC-800. The EIS measurements showed better electrical conductivity of the electrode Cu/NC-700 compared to the other two electrocatalysts.

## Figures and Tables

**Table 1:** Comparison of the catalytic performances of Cu/NC-700 and the similar electrocatalysts reported in literature for the reduction of CO_2_ to liquid products.

Electrocatalyst	Potential (V vs. *RHE*)	Current (mA cm^−2^)	Main Product	FE %	Ref.
Cu NPs	−0.8	−1.0	Ethanol	4	[9]
			Formate	40	
			Acetate	5	
GN/Cu	−0.9	−2.2	Ethanol	9.9	[45]
OD Cu/C	−0.5	-	Ethanol	34.8	[46]
B-doped graphene	−1.4 (vs. S.C.E.)	−1.4	Formate	66	[47]
N-doped G	−0.84	−7.5	Formate	73	[48]
Cu NPs/NG	−1.2	~−1.7	Ethanol	63	[49]
Cu_2_O/ZnO/G	−1.8 (vs. *Ag/AgCl*)	−8.0	N-propanol	30	[50]
Cu_2_O/NGN	−1.0	−7.8	Ethanol	25	[31]
			N-propanol	15	
			Formate	8	
			Acetate	6	
NDD/Si RA	−1.0	−2.0	Formate	14	[43]
			Acetate	77	
Cu/NC-700	−0.8	−4.9	Formate	40.9	This work
Cu/NC-700	−0.8	−4.9	Acetate	16	

## Data Availability

Not applicable.

## Supplementary Materials

The following supporting information can be downloaded at https://www.mdpi.com/article/10.3390/nano13010047/s1: Figure S1: EDS of Cu/NC-700; Figure S2: LSV curves of Cu-NP/NC electrocatalysts in N_2_ and CO_2_ saturated 0.5 M KHCO_3_ electrolyte.; Figure S3: (a) Comparative NMR spectra for the blank electrolyte sample with the internal standard & D_2_O and (b) the sample after the chrono for 2 h at −0.8 V_RHE_ with the internal standard & D_2_O.
