# Supplementary material for: Electrochemical Reduction of CO2 to C1 and C2 Liquid Products on Copper-Decorated Nitrogen-Doped Carbon Nanosheets
Source: Nanomaterials (Basel). 2022 Dec 22;13(1):47. doi: 10.3390/nano13010047 (PMC9824042; doi:10.3390/nano13010047)
Supplement: Supplementary file 1 [file nanomaterials-13-00047-s001.zip › nanomaterials-2072395-supplementary.pdf]

nanomaterials

MDPI

Supplementary Materials

Electrochemical Reduction of CO_2 to C1 and C2 Liquid Products on Copper-Decorated Nitrogen-Doped Carbon Nanosheets

Munzir H. Suliman, Zain H. Yamani and Muhammad Usman *

Interdisciplinary Research Center for Hydrogen and Energy Storage (IRC-HES), King Fahd University of Petroleum & Minerals (KFUPM), Dhahran 31261, Saudi Arabia; munzir.suliman@kfupm.edu.sa (M.H.S.); zhyamani@kfupm.edu.sa (Z.H.Y.)

* Correspondence: muhammadu@kfupm.edu.sa

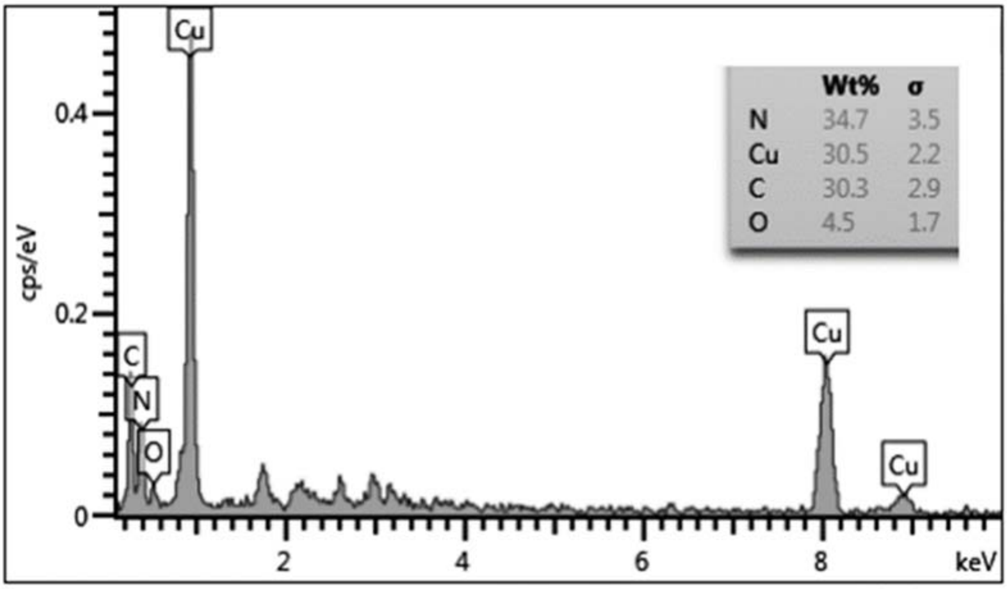

Figure S1. EDS of Cu/NC-700.

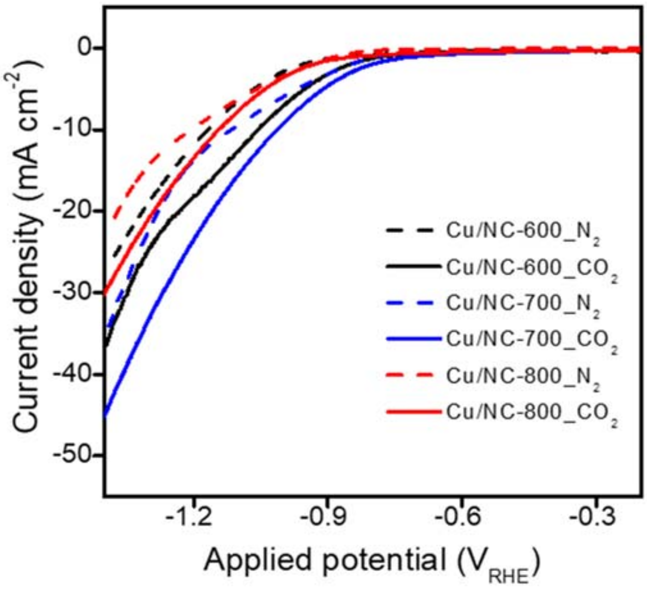

Figure S2. LSV curves of Cu-NP/NC electrocatalysts in N_2 and CO_2 saturated 0.5 M $KHCO_3$ electrolyte.

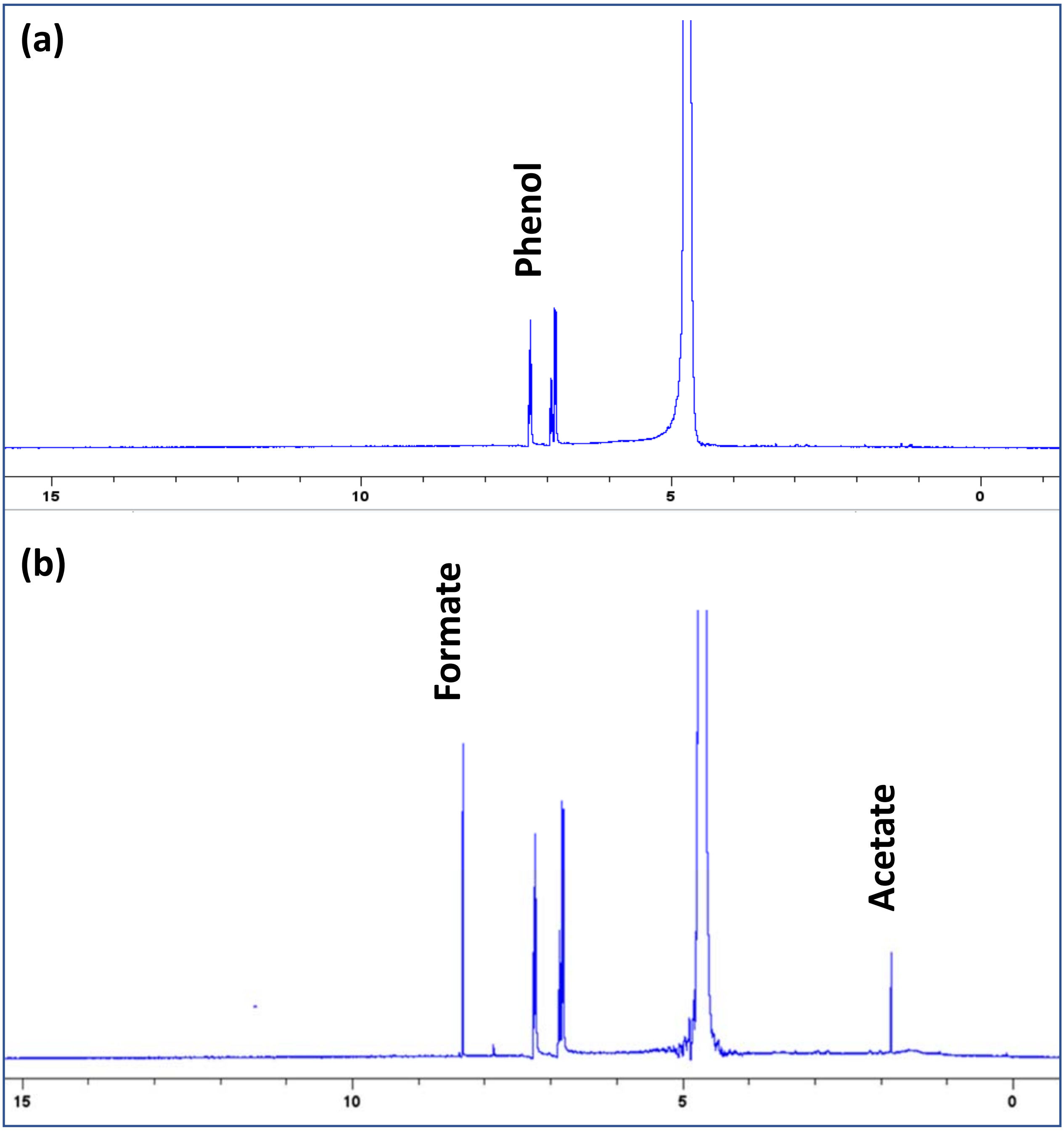

Figure S3. (**a**) Comparative NMR spectra for the blank electrolyte sample with the internal standard & D_2O and (**b**) the sample after the chrono for 2 h at -0.8V_{RHE} with the internal standard & D_2O.
